# The symbolic intersecting ableism and racism scale

**DOI:** 10.3389/fresc.2025.1576357

**Published:** 2025-04-28

**Authors:** Carli Friedman

**Affiliations:** The Council on Quality and Leadership, Towson, MD, United States

**Keywords:** ableism, racism, disability, race, intersectionality, explicit attitudes

## Abstract

**Introduction:**

Intersectionality recognizes and maps the ways oppressions interact and intersect for multiply marginalized people. This framework is a pushing back against the historical approach to discrimination that has taken a “single-axis” view of discrimination, focusing on one single type of oppression, even for people with multiple identities. Little attention has been drawn to intersectionality when it comes to disability, especially related to disability and race.

**Objective:**

In recognition of the intersectional nature of ableism and racism, the aim of this study was to develop and validate the Symbolic Intersecting Ableism and Racism Scale (SIARS).

**Materials and methods:**

We piloted the SIARS with 512 people (July-October 2024) and conducted an exploratory factor analysis to examine the underlying structure of the SIARS.

**Results:**

The SIARS has adequate validity and reliability. Our findings suggest the SIARS is comprised of a complex combination of a denial of continuing discrimination, individualism, and empathy. The findings also indicated many points of contention with the single-axis symbolic ableism scale measure, which examines disability only, further reinforcing the need to measure and attend to intersectionality.

**Conclusion:**

Without doing so, we will never truly be able to dismantle oppression and discrimination, including the ableism disabled people face.

## 1 Introduction

Intersectionality recognizes and maps the ways oppressions interact and intersect for multiply marginalized people (1–3). This framework is a pushing back against the historical approach to discrimination that has taken a “single-axis” view of discrimination, focusing on one single type of oppression, even for people with multiple marginalized identities (1, 4). While sometimes multiply marginalized people may experience discrimination in similar ways to the prototypical member of their identities, they often also face unique experiences due specifically to the intersection of their identities. For example, as Crenshaw (1) notes, while Black women may in some cases experience racism similar to Black men and sexism similar to white women, they also often face unique forms of racism and sexism specifically because of their specific position as Black women.

Similarly, research has found disabled people with other marginalized identities face more discrimination than disabled people without additional marginalized identities (5). Yet, little attention has been drawn to intersectionality when it comes to disability, especially related to disability and race (6, 7). Without attention to intersectionality, disabled people are assumed to all have the same experiences when it comes to discrimination, particularly in a way that is often based on the experiences of white disabled men (6, 8).

However, as recognized by DisCrit—disability studies informed critical race theory—disability and race, as well as their counterparts ableism and racism, are intimately intertwined, feeding off each other (3, 9, 10). Both ableism and racism involve the normalizing of some bodies and minds, and as a result pathologizing of other bodies and minds, via boundaries informed and set by neoliberalism (11–13). For example, the boundaries of what is considered to be a disability have long been informed by race, with non-whiteness sometimes itself believed to be a disability (8, 9, 14–16). Accordingly, many races and ethnicities have been marked as disabled because of their supposed racial inferiority (9, 14, 15); white supremacist laws, policies, and practices are implemented thereafter to deal with these “inferiorities” (9, 17). In contrast, there have also been times when race governs what disability someone is seen to have; for example, when autism was first conceptualized, it was believed that only white people could be autistic and people of color were instead labeled as emotionally disturbed (18, 19).

Moreover, race often shapes how disabled people are treated (20). For example, Black disabled children's behavior is problematized and punished at significantly greater rates than non-Black students (14). Racism can also result in the creation of disability (12, 20, 21); take for example, environmental racism, where people of color are overwhelmingly affected by hazardous conditions and climate change, leading them to acquire disabilities or additional disabilities at greater rates than white people.

### 1.1 Symbolic ableism

In recognition of the intersectional nature of ableism and racism, the purpose of this manuscript is to adapt the Symbolic Ableism Scale (SAS; 22) to examine intersectional attitudes towards disabled people of color. The SAS is a validated explicit attitude measure that examines subtle, yet, often conflicting, attitudes towards disabled people. More specifically, the SAS is comprised of four factors: individualism; recognition of continuing discrimination; empathy for disabled people; and excessive demands. *Individualism*, as deeply informed by neoliberalism, champions not only productivity but also independence, which in turn reframes impairments as disabilities, and denies the reality that we are all interdependent (12, 13, 23, 24). As a result, people are commodified, with their worth based on productivity; doing so helps mask and “naturalize” oppression (25). Core components individualism in the SAS are Protestant work ethic ideology (i.e., the ability to pull oneself up by the bootstraps)—where people are directly responsibility for their own actions—and a just-world ideology—where people are rewarded with good things for good actions. According to individualism, working hard guarantees success and those without success are to blame for their own status. Accordingly, it is believed that disabled people can “overcome” their disabilities if they just try hard. People often use individualism to justify their biases and social inequities (26). Similarly, the factor *excessive demands* suggests disabled people demand too much, including special favors and overuse of the welfare system, including in ways they are not necessarily deserving of (22). While these two themes represent negative views of disability, the other two themes—*recognition of continuing discrimination* and *empathy for disabled people*—recognize the unfair treatment disabled people experience; these attitudes are still informed by ideas of “deservedness,” including pity, and paternalism, which can be expressions of ableism as well.

Research has found a relationship between symbolic ableism and social dominance orientation—a favoring of social hierarchies—including anti-egalitarianism (26, 27). In fact, Raoul (26) found symbolic ableism plays a mediating role in the relationship between social dominance orientation and people's support for health policies, including Medicare for all, unlimited paid sick leave, and private health insurance.

The SAS was itself developed based on the symbolic racism scale (SRS), which was developed in the 1970s to examine attitudes towards Black people and then revised multiple times over the years (28, 29). To do so, when the SAS was developed, all versions of the SRS were reviewed (28–31), and revised to focus on disability according to research on disability prejudice and ableism (e.g., 17, 32–37). While the themes *individualism* and *excessive demands* in the SAS somewhat parallel the SRS, the other two themes of the SAS contrast with the SRS in that for disabled people, there was an acknowledgement of discrimination, whereas in the SRS there was a denial of continued racial discrimination (22, 28). Moreover, whereas in the SAS participants had empathy for disabled people, in the SRS, participants believed Black people had more undeserved outcomes and advantages (22, 28).

These points of contention between symbolic ableism—the SAS—and symbolic racism—the SRS—as well as a growing recognition that single-axis measures of discrimination do not adequately capture all aspects of oppression, suggest a need to develop an intersectional tool that looks at how ableism and racism interact. For these reasons, the aim of this study was to develop and validate the Symbolic Intersecting Ableism and Racism Scale (SIARS). To do so, we piloted the SIARS with 512 people and conducted a factor analysis to examine the underlying structure of the SIARS.

## 2 Materials and methods

### 2.1 Participants

This study was part of a larger study examining the intersection between ableism and racism, both explicitly and implicitly. For the larger study, after institutional review board (IRB) approval, we recruited (July 31 to October 3, 2024), via random stratified sampling, adults in the United States from National Institute of Health's (NIH's) ResearchMatch. ResearchMatch is a national health volunteer registry that was created by several academic institutions and supported by the United States National Institutes of Health as part of the Clinical Translational Science Award (CTSA) program. While a total of 536 people participated in the larger study, for this study we removed (*n* = 24) people who did not answer all of the SIARS questions, resulting in a final sample size of 512 people.

The average age of participants was 43.11 years old (Table 1). Most participants were cisgender women (66.60%), white (76.37%), and straight (68.70%). In total, 39.84% of participants identified as nondisabled and 60.16% of participants identified as disabled, with mental health disabilities being the most common (40.82%). The most common education level was bachelor's degree. While income was relatively evenly distributed across categories, the most common was $50,000 to $74,999 (21.41%). Participants lived in 46 different states (all states except Alaska, Kentucky, Montana, Wyoming), the District of Columbia, and Puerto Rico, with an average of 10.67 participants (SD = 11.30) per state/territory/capital.

**Table 1:** Sociodemographics (*n* = 512).

Characteristic	*n*	%
Age [*M* (SD)]	43.11 (16.00)	
Disability		
Nondisabled	204	39.84%
Disabled^a^	308	60.16%
Mental health disability	209	40.82%
Chronic illness	114	22.27%
Intellectual and/or developmental disability	60	11.72%
Physical disability	37	7.23%
Sensory disability	35	6.84%
Other disabilities	32	6.25%
Gender^a^		
Cisgender woman	341	66.60%
Cisgender man	129	25.20%
Nonbinary	22	4.30%
Genderqueer	12	2.34%
Agender	5	0.98%
Trans masc	5	0.98%
Trans woman	3	0.59%
Trans man	1	0.20%
Other	8	1.56%
Race^a^		
White	391	76.37%
Latine	75	14.65%
Asian	68	13.28%
Black	33	6.45%
Indigenous	27	5.27%
Middle Eastern	15	2.93%
Native Hawaiian or other Pacific Islander	6	1.17%
Other	6	1.17%
Sexual orientation^a^ (*n* = 492)		
Straight	338	68.70%
Bisexual or pansexual	83	16.87%
Gay or lesbian	36	7.32%
Aromantic	29	5.89%
Asexual	25	5.08%
Other	10	2.03%
Education level (*n* = 470)		
High school graduate (or equivalent)	51	10.85%
Associate degree	51	10.85%
Bachelor degree	194	41.28%
Graduate degree	174	37.02%
Income (*n* = 439)		
Less than $25,000	78	17.77%
$25,000 to $34,999	49	11.16%
$35,000 to $49,999	46	10.48%
$50,000 to $74,999	94	21.41%
$75,000 to $99,999	74	16.86%
$100,000 to $149,999	61	13.90%
$150,000+	37	8.43%

^a^ Note: Participants could select more than one subcategory.

### 2.2 Measures

#### 2.2.1 SIARS

Content for the SIARS was adapted from the SAS, changing the focus from “disabled people” broadly to “disabled people of color” specifically. The measure included the following variables (presented to the participants in random order):

1. Even if disabled people of color try hard, they often cannot reach their goals (Reverse keyed).
2. Even if disabled people of color are ambitious, they often cannot succeed (Reverse keyed).
3. If disabled people of color work hard they almost always get what they want.
4. Hard work offers little guarantee of success for disabled people of color (Reverse keyed).
5. Any disabled person of color who is willing to work hard has a good chance of succeeding.
6. Discrimination against disabled people of color is no longer a problem in my country.
7. If disabled people of color would just try harder they would be as well off as nondisabled people.
8. Disabled people of color are demanding too much from the rest of society.
9. Disabled people of color should stay hidden.
10. Most disabled people of color who don't get ahead should not blame the system; they really have only themselves to blame.
11. Over the past few years disabled people of color have gotten less than they deserve (Reverse keyed).
12. It is easy to understand the anger of disabled people of color in this country (Reverse keyed).
13. Disabled people of color complain too much about their situation and society.

Participants rate their level of agreement with each above statement on a seven-point Likert scale from strongly disagree to strongly agree. As with the SAS, scores for each Likert value range from 0 to 1 depending on their location on the scale (e.g., strongly disagree = 0, neither agree nor disagree = 0.5, strongly agree = 1).

#### 2.2.2 Other explicit measures

To examine convergent validity, we also included two additional explicit measures so they could be compared to the SIARS. The first asked participants their strength of preference for nondisabled white people relative to disabled people of color on a seven-point Likert scale with value equivalents of −3 to 3 [e.g., I strongly prefer disabled people of color over nondisabled people (−3)], with more positive scores representing more preference for nondisabled white people.

The second additional measure asked participants to rate how warm or cold they feel towards nondisabled white people and how warm or cold they feel towards disabled people of color as separate questions on seven-point Likert scales [e.g., very cold (−3), neutral (0), moderately warm (2), etc.], with value equivalents of −3 to 3. The difference in their responses to these two questions (i.e., the two questions subtracted from each other) was used as their degree of warmth for nondisabled white people relative to disabled people of color, with more positive scores representing more warmth for nondisabled white people.

### 2.3 Procedure

After completing the informed consent, participants first completed the Intersecting Disability and Race Attitudes Implicit Association Test (IDRA-IAT), which is the subject of a separate study (38). Next, participants completed the SIARS and then the other two explicit measures. Finally, participants answered questions about their sociodemographics. They were then thanked for their participation and compensated with a $15 gift card for their time.

### 2.4 Data screening and analysis

We used SPSS 27 for this analysis. Data were collected from 512 people to develop the SIARS. This satisfied the minimum amount of data needed for factor analyses, with a ratio of over 39 cases per variable (39). First, we reverse keyed the applicable SIARS items. We then excluded outliers (40, 41). Finally, we ran an EFA with the 13 SIARS variables in the measure to compute compositive scores for the factors underlying the model; promax rotation was used for the EFA. One variable (“It is easy to understand the anger of disabled people of color in this country”) did not sufficiently load onto any of the factors so it was removed, one variable (“even if disabled people of color try hard they often cannot reach their goals”) did not have a sufficient commonality so was removed, and then one variable (“Over the past few years disabled people of color have gotten less than they deserve”) became cross-loaded between two factors so was removed. The EFA was then re-run with promax rotation without these variables—with the other 10 SIARS variables—for the final model presented below. Pearson correlation was used to examine the relationship between the SIARS and the other two explicit measures of preference and warmth. Finally, we ran descriptive statistics.

## 3 Results

**Figure 1:**
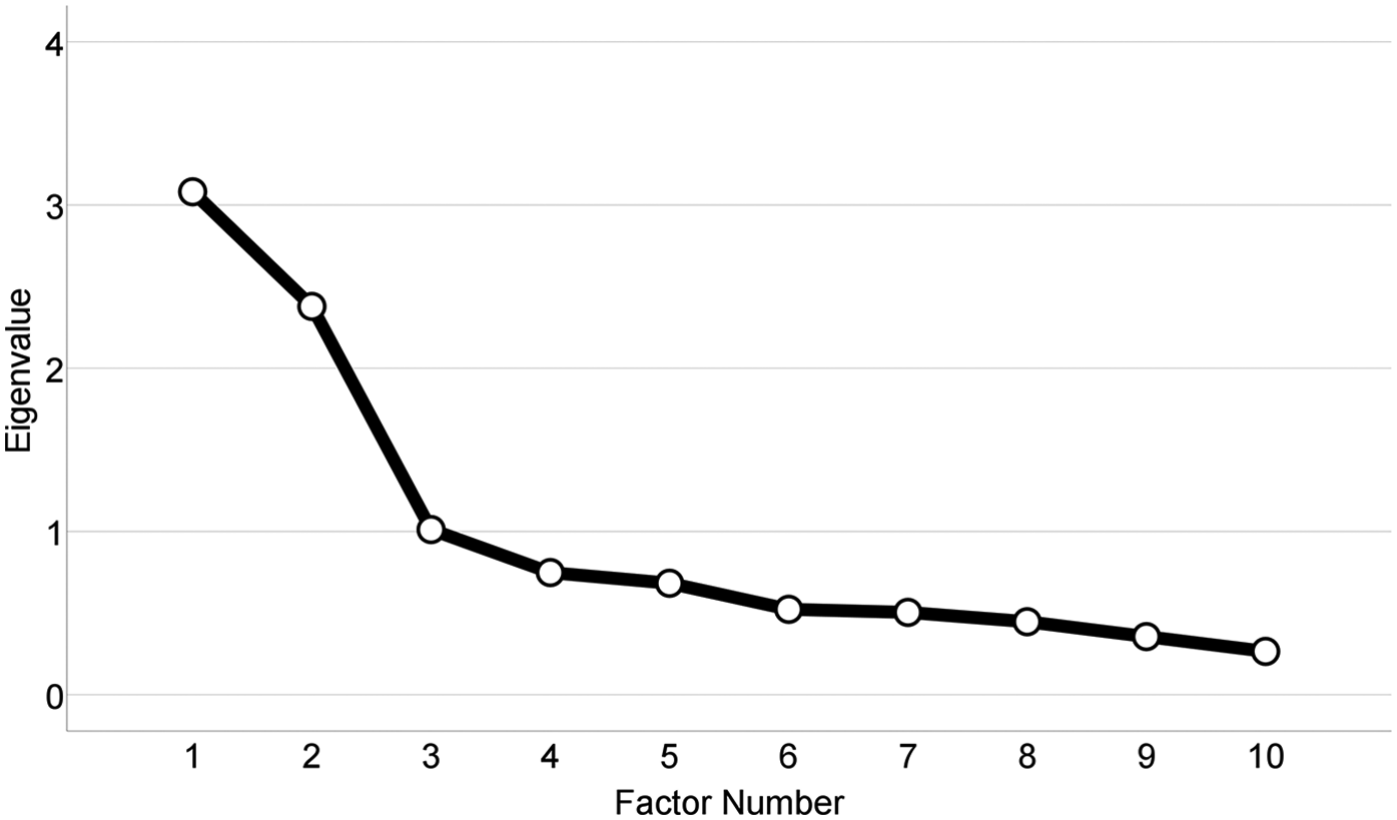
Scree plot (Alt text: the screen plot indicating the number of factors and their eigenvalues. The first three factors have eigenvalues of above one, with the first and second factors having the highest eigenvalues. Factors 4 through 12 have eigenvalues below one and gradually decline as the factor number increases).

Sampling adequacy using the Kaiser-Meyer-Olkin measure was 0.76 and Bartlett's test of sphericity was found to be significant [*χ*^2^_(45)_ = 1,229.67, *p* < 0.001]. The findings from the EFA revealed the 10 indicators loaded into three factors with eigenvalues that exceeded 1.00. These three factors explained a cumulative variance of 64.72%, with the first factor explaining 30.81% of variance, the second 23.79%, and the third 10.12% (Table 2; Figure 1).

**Table 2:** Factor loadings and communalities.

Item	Factor			Communality (*h*^2^)
	Denial of continuing discrimination	Individualism	Empathy	
Discrimination against disabled people of color is no longer a problem in this country.	0.86			0.77
If disabled people of color would just try harder they would be as well off as white nondisabled people.	0.69			0.50
Disabled people of color are demanding too much from the rest of society.	0.75			0.57
Any disabled person of color who is willing to work hard has a good chance of succeeding.		0.80		0.56
Even if disabled people of color are ambitious they often cannot succeed (R).		0.61		0.46
Hard work offers little guarantee of success for disabled people of color (R).		0.67		0.51
If disabled people of color work hard they almost always get what they want.		0.44		0.36
Most disabled people of color who don't get ahead should not blame the system; they really have only themselves to blame.			0.72	0.55
Disabled people of color should stay hidden.			0.52	0.36
Disabled people of color complain too much about their situation in society.			0.45	0.41

Note: (R), reverse coded.

The three factors were examined and compared to the SRS and SAS to determine themes. The first SIARS factor, *Denial of Continuing Discrimination*, included questions 6, 7, and 8. The second SIARS factor, *Individualism*, included questions 2, 3, 4, and 5. The third SIARS factor, *Empathy*, included questions 9, 10, and 13. Cronbach's alpha was 0.80 for *Denial of Continuing Discrimination* (factor 1), 0.74 for *Individualism* (factor 2), and 0.59 for *Empathy* (factor 3). Cronbach's alpha across the scale was 0.70.

The SIARS was significantly correlated with our two other explicit measures: preference for nondisabled white people relative to disabled people of color (*r* = 0.23, *p* < 0.001), and warmth for nondisabled white people relative to disabled people of color (*r* = 0.22, *p* < 0.001). While these correlations were not large, this is to be expected given both additional explicit measures were rather one-dimensional and do not measure the same complexity or level of detail as the SIARS.

Across the scale, participants had a mean score of 0.20 (SD = 0.12). Ranges were created using the percentiles of participants' averages across the 10 variables of the SIARS. According to the findings, SIARS scores of 0–0.101 (25th percentile) are considered to have little to no intersecting disability and race bias, scores of 0.1011–0.200 slight intersecting bias, scores of 0.2001–0.284 (up to 75th percentile) moderate intersecting bias, and scores of 0.2841 + strong intersecting bias.

Descriptive data for the SIARS factors and variables are available in Table 3. The mean score of participants on factor 1 suggests that participants continue to strongly discriminate against disabled people of color (*M* = 0.32). Means on factor 2 suggest participants are moderately biased against disabled people of color when it comes to individualism (*M* = 0.28). Finally, the mean for the third factor suggests participants have empathy and low levels of bias for this factor (*M* = 0.07).

**Table 3:** Descriptive statistics.

Indicator	*M*	SD
Factor 1: Denial of continuing discrimination	0.32	0.26
Discrimination against disabled people of color is no longer a problem in this country.	0.40	0.35
If disabled people of color would just try harder they would be as well off as white nondisabled people.	0.32	0.29
Disabled people of color are demanding too much from the rest of society.	0.25	0.27
Factor 2: Individualism	0.28	0.20
Any disabled person of color who is willing to work hard has a good chance of succeeding.	0.45	0.31
Even if disabled people of color are ambitious they often cannot succeed (R).	0.30	0.29
Hard work offers little guarantee of success for disabled people of color (R).	0.25	0.30
If disabled people of color work hard they almost always get what they want.	0.18	0.20
Factor 3: Empathy	0.07	0.09
Most disabled people of color who don't get ahead should not blame the system; they really have only themselves to blame.	0.04	0.09
Disabled people of color should stay hidden.	0.04	0.09
Disabled people of color complain too much about their situation in society.	0.16	0.20

Note: Higher scores represent more bias. (R), reverse coded.

## 4 Discussion

The aim of this study was to pilot and validate a measure of intersecting ableism and racism—the SIARS. Our factor analysis revealed the final SIARS to be:

1. Discrimination against disabled people of color is no longer a problem in this country.
2. If disabled people of color would just try harder they would be as well off as white nondisabled people.
3. Disabled people of color are demanding too much from the rest of society.
4. Any disabled person of color who is willing to work hard has a good chance of succeeding.
5. Even if disabled people of color are ambitious, they often cannot succeed (Reverse keyed).
6. Hard work offers little guarantee of success for disabled people of color (Reverse keyed).
7. If disabled people of color work hard they almost always get what they want.
8. Most disabled people of color who don't get ahead should not blame the system; they really have only themselves to blame.
9. Disabled people of color should stay hidden.
10. Disabled people of color complain too much about their situation in society.

### 4.1 Denial of continuing discrimination

Factor one, *Denial of Continuing Discrimination*, is one of the points of contention between the SIARS and the SAS. In the SAS, people *Recognized Continuing Discrimination* and scored relatively low levels of bias in this factor. In contrast, in the SIARS, when the focus is specifically on disabled people of color, rather than disabled people more broadly, people were more likely to reinforce discrimination and score as more biased. As such, it appears people are more likely to “recognize” discrimination against disabled people more broadly but deny it for disabled people of color. We theorize this is likely because when people are presented with “disabled people,” even though it is technically across races, people are likely primed to think of the prototypical disabled person, likely a white disabled person. For example, when Goff and Kahn (42) asked people to think of targets of racism, they imagined men, and when people were asked to think about the targets of sexism, they thought about white women. Single-axis measures which only focus on race, gender, or disability, may still implicitly lead people to think about intersections, thus reinforcing the need for intersectionality, including to examine how ableism may operate differently for non-white races.

### 4.2 Individualism

Individualism is a factor in both the SAS and SIARS, reflecting the important role individualization of disability and “bootstrapping”—being responsible for ones' own outcomes and being able to achieve anything with hard enough work—plays in both ableism and racism. In fact, individualization of disability, sometimes also referred to as the medical model of disability—where disability is framed as a medical problem located in the individual disabled person—and ableism are intertwined and nearly impossible to separate as individualization is not only an expression of ableism, but ableism also reinforces individualization. Moreover, individualism is used to reinforce, justify, and “naturalize” white supremacy (15). The key role neoliberal individualism play in ableism and racism are why several key principles of disability justice include recognizing and pushing back against capitalism's commodification and marginalization of disabled people, especially disabled people of color (21).

It is important to note that while *individualism* plays significant roles in both the SAS and SIARS, it is more dominant in the SAS, which examines ableism only, explaining a larger proportion of variance and with larger factor loadings and commonalities, than the SIARS, which examines the intersection between ableism and racism. Rather, in the SIARS, *Denial of Continuing Discrimination* is the most prominent theme, instead of *Individualism*. While more research is needed to examine these trends, the change in directionality of factor one between the SAS and SIARS may play an important role in these findings. It may be that if people are somewhat recognizing discrimination exists (in the SAS), they are more likely to focus on individual failings (e.g., not working hard enough) as the cause of disabled people's status. Whereas, if people are denying discrimination exists and effects disabled people of color (in the SIARS), they are more likely to reinforce discriminatory attitudes, as well as individualize.

### 4.3 Empathy

The final factor of the SIARS was empathy. While in both the SAS and SIARS, this factor is unique to measures of symbolic attitudes that focus on disability—it is not in the SRS—likely because of the unique role of disability, including when it comes to pity. While empathy can be a good thing, empathy for disabled people is often driven by assumptions that disabled people are more childlike or innocent, that they are less capable, and that they are deserving of help, all of which are problematic stereotypes that result in infantilization and paternalism (43–46).

However, unlike the SAS, in the SIARS empathy did not include “It is easy to understand the anger of disabled people of color in this country,” which was excluded from the scale altogether due to not loading onto any of the factors. In fact, when examined outside of our main analysis, people had statistically significantly more bias on this indicator when it included disabled people of color in the SIARS [*M*(SD) = 0.28 (0.27)] than when it did not explicitly include people of color on the SAS [*M*(SD) = 0.22 (0.28); *t*_(665)_ = 2.51, *p* = 0.01]. As such, these findings suggest people may have more difficulty understanding the anger of disabled people specifically when they are disabled people of color. In fact, the concept of “anger” in and of itself is a racialized one, intertwined with ones' race, with stereotypes suggesting Black people are more angry or quicker to anger, and their behaviors being interpreted as angry more frequently (47–50).

### 4.4 Limitations and suggestions for future research

Several limitations should be noted when interpreting this study's findings. While random sampling was used, given participants all were registered for ResearchMatch and all opted in to participate, there is a chance of self-selection bias. The sample was not representative. For example, the majority of participants were white. This may have impacted the results as people of color could have more positive attitudes due to ingroup favoritism (5, 51). Since this was an online survey, it might not have been accessible for all disabled people depending on their impairments or support needs. It should also be noted that the Cronbach's alpha for *Empathy* is slightly below the common threshold, this is likely partially caused by only having three items in the factor (52, 53) and also due to our removal of outliers—when outliers are included the Cronbach's alpha for *Empathy* is 0.73. Moreover, the analysis met other criteria for factor retention (54, 55) and had reliable loadings (56), in addition to the overall scale meeting the acceptable threshold. That being said, future research should examine if adding additional items to this factor improves reliability.

While the SIARS measures across race and across disability, it is important to note that both racism and ableism can function differently across different races and disabilities, including the intersection of the two. As such, future research should explore these differences, including the applicability and potential for developing further versions of the SIARS that examine interactions between more specific identities, and tease out any potential differences in measures.

In addition, while in this study our aim was to move the SAS beyond a single-axis measure towards an intersectional one, particularly one that focused on the intersection between ableism and racism, the SIARS does not cover all intersections. For example, gender too informs disability, in addition to how disability and race intertwine. As such, the development of additional tools to measure intersectionality would be beneficial. In fact, rather than a conclusion, we like to think of the SIARS as a starting point, that creates new directions for the exploration of how discrimination and oppression work, and, by extension, how to successfully dismantle them. In addition, given symbolic racism and symbolic ableism research have separately found symbolic attitudes can help predict people's policy preferences (26, 28), the SIARS can be used to further examine intersecting structural oppression, which plays an important role in the lives of disabled people of color. In fact, there are many opportunities for future research about the impact intersectional implicit attitudes have on the lives of disabled people of color, in order to ultimately change them. For example, the SIARS could be used to explore how health care professionals' attitudes impact their interactions with disabled people of color and, as a result, the outcomes of disabled people of color. Given the differences between the SIARS and SAS, especially the denial of continuing discrimination in the SIARS, in contrast to a recognition of discrimination in the SAS, more nuanced anti-ableist and anti-racist education and interventions are needed, particularly to disrupt the ways people think about and understand discrimination towards disabled people of color. The SIARS could even be used to test the effectiveness of these initiatives.

## 5 Conclusion

The concept of intersectionality recognizes and reminds us that single-axis measures of oppression are not sufficient, especially for examining the impact on people with multiple marginalized identities (1–3). Yet, there has been less attention to disability when it comes to intersectionality (6, 7). As such, in this study, we sought to develop a new measure that can be used to examine the intersection between ableism and racism, the SIARS. Our findings suggest the SIARS is comprised of a complex combination of denial of continuing discrimination, individualism, and empathy. The findings also indicated many points of contention with the single-axis SAS measure, which examines disability only, further reinforcing the need to measure and attend to intersectionality.

The SIARS follows many of the DisCrit's principles (9), including the push to move away from single-axis approaches to identity, and recognizing that prototypical-based measures likely distort disabled people's experiences via the privileging of white disabled people. In addition, DisCrit's tenants recognize that ableism and racism are intersectional, and that intersectionality not only enforces normality and its (socially-created) boundaries, but also helps naturalize ableism and racism (9). This is mirrored both in the SIARS' intersectional approach and its emphasis on individualism, and the boundaries set forth and reinforced by neoliberalism. Through its measures, the SIARS also focuses on historic and current day discrimination, including systemic barriers; doing so also aligns with DisCrit's emphasis on the ways laws and history have been leveraged to oppress disabled people. The final tenant of DisCrit is that “DisCrit requires activism and supports all forms of resistance” (9). While we recognize the creation of a measure itself will not create change, it is our hope that it is a tool that can be used to learn more about how intersectional discrimination operates, in order to help support this change-making. Without attending to intersectionality, we will never truly be able to dismantle oppression and discrimination, including the ableism disabled people face.

## Data Availability

The datasets presented in this article are not publicly available because this was not a condition of participant consent. Further inquiries can be directed to the corresponding author.
